# Basic Public Health Service Utilization by Internal Older Adult Migrants in China

**DOI:** 10.3390/ijerph18010270

**Published:** 2021-01-01

**Authors:** Dan Tang, Jiwen Wang

**Affiliations:** 1 Center for Population and Development Studies, Renmin University of China, Beijing 100872, China; dantang@ruc.edu.cn; 2 Institute of Gerontology, Renmin University of China, Beijing 100872, China; 3 Population Studies and Training Center, Brown University, Providence, RI 02912, USA

**Keywords:** health service use, migrant older adults, physical examinations

## Abstract

Since 2009, the Chinese government has launched a basic public health services (BPHS) equalization program to provide the same BPHS to all the citizens. However, utilization of BPHS among older migrants is still low. The purpose of this paper was to explore the determinant individual and contextual factors of older migrants’ utilization of BPHS, and to provide suggestion for the government to improve BPHS utilization. Based on Andersen’s model of health services use, data from the China’s Regional Economic Statistics Yearbook 2014 and National Health and Family Planning Dynamic Monitoring Survey on Migrant Population 2015 were analyzed using a hierarchical random intercept model for binary outcomes. Results showed that the percentage of migrant older adults receiving free physical examinations, which is an important item of BPHS, was 36.2%. Predisposing (education, hukou, living duration in the host city, and scope of migration), enabling (health insurance and social networks), and need (self-rated health and chronic conditions) factors of individuals’ characteristics had significant impact on the use of BPHS. The proportions of both migrant children enrolled in public schools and people with established health records had a positive impact on an individual’s chance of receiving free physical examinations. These findings suggest that economic development and improvement at the level of the city’s health resources cannot effectively improve access to BPHS by older adult migrants. Instead, the driving force appears to be supportive policies for the migrant population.

## 1. Introduction

Over the past and recent decades, China has gained an immense internal migrant community. The number of migrants increased from an estimated 230 million in 2011 to 241 million in 2018 [1]. Since 2000, the population of migrant older adults has increased with the trend of population aging and family migration. According to the figures of the National Health Commission, the proportion of migrants over the age of 60 years is around 7.2%, which is close to 18 million [2]. Due to the “hukou”, a rigid household registration system which regulates population distribution, older adult migrants, however, often possess poorer socioeconomic characteristics and are notoriously marginalized in China [3]. Under the “hukou” system, migrants do not share many of the privileges of the local residents [4]. The socioeconomic status of the older migrants is poorer than that of local residents because they usually arrive in the cities in their later life and thus, encounter more difficulties in adapting to a new life [5,6]. They are not entitled to the same social benefits as the local residents, and are less likely to have comprehensive access to healthcare services, comfortable housing, and fair life conditions [7,8]. Compared with local counterparts, older adult migrants report poorer physical and mental health and are more vulnerable to long-term health problems [3]. Therefore, access to health services is important for older adult migrants, which can provide protection for their health.

In recent years, the central government and local authorities have paid increasing attention to migrants’ health as part of a wider policy agenda towards more inclusive social and economic development [9]. In 2009, the government issued a landmark health policy to promote universal access to health services and launched the National Basic Public Health Service Program (BPHS). BPHS aims to provide free of charge essential health services for all citizens at the point of care [10]. There are currently 14 items in the BPHS services package, of which a vital part is geriatric health services [11,12]. According to the BPHS, residents who are aged 65 years or above and in the catchment area are eligible to receive geriatric health management services, which include lifestyle and health status evaluation, physical examination, auxiliary examination, and health guidance [13]. Since the most important goal of the most recent healthcare reform was to provide equitable and accessible public health services to every Chinese citizen, all services in the BPHS package were provided to all the residents in the catchment area, including non-registered people (i.e., those without local hukou) who had lived there for at least 6 months [14]. In keeping with the policy drive towards equalization of the BPHS, the issue of access to health care for migrants was given more attention. The Chinese government promoted access to BPHS for migrants using multiple measures [10].

There are still substantial barriers preventing older adult internal migrants from accessing BPHS. Chinese internal migrants, including those aged over 60 years, have low utilization of BPHS compared with residents with local hukou [15]. The source of the obstacles has both contextual and individual elements. Many local health departments have insufficient capacity to provide all internal migrants with the full BPHS package. Although specific health programs for internal migrants have been developed, fiscal transfers to local authorities for public service provisions are generally based on the number of hukou residents. Therefore, many counties and municipalities may lack sufficient resources to implement public health programs, especially in cities where migrants account for a large proportion of residents and the local economy is less developed [16,17]. Furthermore, family factors, instead of economic factors, have shown to be the main migration motivation for older adults [18]. The labor participation rate of older adult migrants is much lower than that of working-aged migrants, and they are less likely to contribute to the economic and social development of cities. As a result, compared with the migrants of younger age groups, policy makers and government administrators pay less attention to older adult migrants [3]. The policies that are aimed to reduce inequities in access to health services, such as the BPHS, are targeted more towards working-age migrants and their children. Few policies pay specific attention to migrant populations aged 60 years and above [10,14]. Apart from the barriers that originate from the government, there are obstacles that affect access to BPHS that are borne by the migrants. The lack of information about the BPHS, insufficient knowledge about health, and negative attitudes toward health preventive behaviors have resulted in difficulties for older migrants to utilize the BPHS [15,19]. As a result, the situation is the most serious for older adult migrants compared with any other migrant age group. Inadequate use of BPHS has a negative impact of older adult migrants’ health. For example, lack of physical examination may result in the failure to detect hidden disease in early stage; lack of chronic disease management by the community doctors may exacerbate the symptoms.

The health of older adult migrants is an indispensable component of the Healthy China 2030 Plan [20]. The use of the BPHS is critical to prevent disease and provide health promotion measures for older adult migrants in China. Systematic work on the use of the BPHS among Chinese internal older adults is far from adequate. To our knowledge, there is little information on the utilization of health services among Chinese older adult migrants. The few studies that have investigated the internal migrants’ utilization of public health services, and most of them were limited in labor aged group [14,15,21], and only one study pay attention to the older migrants [22]. Moreover, they mainly emphasized the demographic, socioeconomic and migration factors in the individual level but did not examine the influence of contextual characteristics [23,24]. The utilization of health care among older adult migrants is vital for older migrant individuals, community services and government managements. We need to know more about determinants in both individual and contextual levels of BHPS utilization among Chinese older adult migrants. Moreover, China has implemented a strict household registration system since the 1950s, and this rural/urban dichotomy system has resulted in sharp social and economic disparities between rural and urban populations [25]. There are large differences in health awareness, health status, and health habits between migrant who come from rural and urban areas [26,27,28], the rural-urban disparity should not be neglect.

The aim of this study is to explore the contextual and individual determinants of Chinese older adults migrants’ utilization of BPHS, and to provide evidence and policy recommendations from contextual and individual levels for promoting the utilization of BPHS among older adult migrants and achieving the goal of equalization of the health services plan. As a developing country with the world’s largest internal migrant and aging population, the development of BPHS is facing great challenges. The situation in China will serve as an important reference for other countries with large migrant population.

## 2. Materials and Methods

### 2.1. Data

Cross-sectional data were mainly from the 2015 National Migrants Population Dynamic Monitoring Survey (MDMS), which was conducted in May 2015 by the China Population and Development Research Center and commissioned by the National Health and Family Planning Commission. The survey adopted a stratified, three-stage probability proportional to size sampling (PPS) method. The annual national data on migrants from each of the 32 provincial units in 2014 was considered as the basic sampling frame. It surveyed a nationally representative sample of the migrant population aged 15 years and above who had been living in their host cities for more than one month but had not registered hukou in their local district (commonly referred to as a floating population) [3]. All participants provided informed consent. A health service module for older adults over 60 years old was designed in the 2015 survey only, which provided us with an opportunity to examine the associations between individual and contextual factors and BPHS utility in the older adult migrants in China.

**Figure 1:**
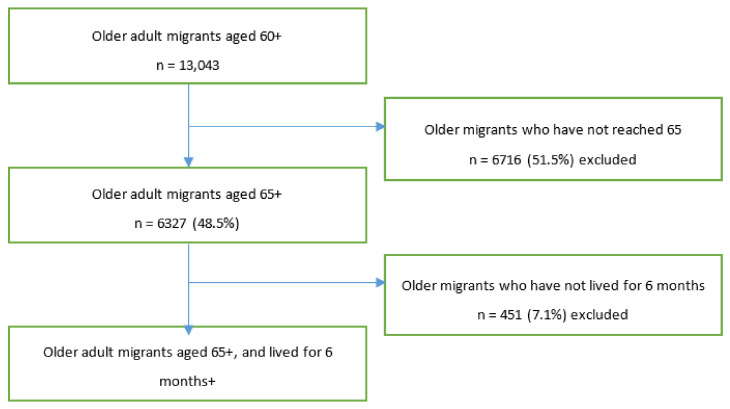
Sampling procedure.

Given our target population of people who use geriatric health services in the service package of BPHS [13], we restricted our sample to participants aged 65 and above who had been living in their area of residence for at least 6 months. The age restriction resulted in about 51.5% (n = 6716) of the migrant older adults to be excluded from the initial sample (n = 13,043). From the remaining sample, the 6-month residence restriction resulted in 7.1% (n = 451) to be excluded from the 65 years or older subsample (n = 6327). The final sample size was 5876, located in 297 cities (Figure 1. Sampling procedure).

Several city-level variables were used to estimate the relationship between contextual characteristics and the use of BPHS. Each city’s socioeconomic indicators were obtained from the 2014 China Regional Economic Statistical Yearbook. The city’s indicators that were related to the migrant population were computed from the MDMS 2013 and 2015.

### 2.2. Analytic Framework

**Figure 2:**
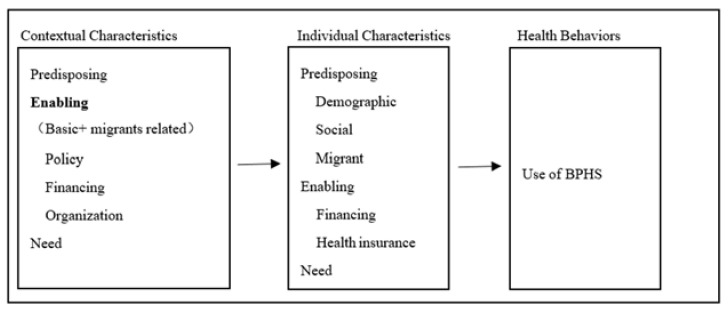
The simplified Andersen’s model of health services use.

To determine the utilization of BPHS among older adult migrants, we used a simplified version of Andersen’s model of health services use (Figure 2) [29].

It is a well-validated theoretical framework aimed at understanding determinants of health services utilization, which stresses that improving access to care is best accomplished by focusing on contextual as well as individual determinants [15]. According to the model, the health behaviors (including personal health practices, process of medical care, and use of personal health services) is determined by contextual and individual characteristics, both of which include predisposing, enabling and need factors. We will stress the impacts of contextual enabling factors, which are the conditions of a group units (size from family to nation) that facilitate or impede use of health services. Compared to predisposing and need factors, the contextual enabling factors are more important and operational for the government [30]. Contextual enabling factors can be divided into policy (the authoritative decisions made pertaining to health or influencing the pursuit of health), financing (an array of contextual measures that suggest resources potentially available to pay for health services) and organization (the amount and distribution of health services facilities and personnel as well as how they are structured to offer services). We were not only interested in the basic characteristics of the enabling factors of the city, but also in the cities’ corresponding characteristics that relate to the migrant population. As for the individual characteristics, we will test all the three factors (predisposing, enabling and need) as previous researches [15].

### 2.3. Measures

#### 2.3.1. Dependent Variable

We chose participation in the free annual physical examination as the indicator for utilization of BPHS among older adult migrants. There were two variables which measured the utilization of BPHS: having an established health record and participating in the free physical examination. Compared with establishment of health record, previous studies have demonstrated that periodic physical examination is a more important and effective measure of illness prevention and health promotion through early detection and timely treatment [27]. Free physical examination is measured by asking the older adult migrants: “Have you ever participated in a free physical examination (excluding the examination due to illness) organized by a community health service station/center in the past year?” The question is a binary variable that required a ‘yes’ or ‘no’ response.

#### 2.3.2. Contextual Characteristics

Since we were concerned about migrant older adults, for the context characteristics, we not only paid attention to the general situation of the city, but also to the cities’ features that relate to the migrant population, as shown in Table 1. All the contextual characteristic variables were collected at the level of the prefectural city.

##### Policy

Since the policy of free annual physical examinations for older adults in the BPHS is consistent throughout the country, it is a constant across different cities and is therefore not included in the quantitative analysis. We use the proportion of migrant children enrolled in public schools in each city who receive compulsory education as the migrant-related policy indicator, which was used in previous study [31]. We believe that this indicator can reflect a city’s acceptance of the migrant population, especially of those of non-working age [32,33]. Both compulsory education and BPHS are national basic public services in China [32,34] and are free for all residents within the jurisdiction, irrespective of hukou status. Thus, enrolment of migrant children in public schools and the utilization of free physical examinations for older adult migrants should be closely related. If it is easier to enroll migrant children in public schools in a city, there will be fewer obstacles for migrants to access public services in that city, which means that the city’s acceptance of migrants of any age group will be higher. The percentage of migrant students enrolled in public schools is calculated based on the data from the MDMS 2013. The percentage of migrant students enrolled in public schools is positively correlated with the years of living duration in the host city (r = 0.039) and the conceived identity of being the local people (r = 0.205) from 2017 survey data (*p* < 0.001).

##### Financing

The general financing feature is evaluated by the gross regional product (GRP) of the city and was obtained from the 2014 China Regional Economic Statistical Yearbook. The migrant-related contextual financing characteristics were measured by the household income of the migrant population per capita in each city, which was calculated from the MDMS 2015.

##### Organization

The general organizational elements of a city were measured by the number of doctors per thousand people, which reflects the health service resource of the city [35]. The data was obtained from the 2014 China Regional Economic Statistical Yearbook. The migrant-related organization factor was measured by the proportion of health record establishment among the city’s migrant population of all age groups, which reflects the efforts made by the host city’s health institutions to establish health records for its migrants. This data was obtained from the MDMS 2015.

#### 2.3.3. Individual Characteristics

Predisposing, enabling, and need factors at the individual level were included in our models (see detailed variable settings in Table 2. Individual and contextual variables, and the associations with free examinations). Predisposing factors included socio-demographic variables (age, gender, current marriage status, education, and hukou registration type) and migration characteristics, which included living duration in the host city and scope of migration distance. Current marriage status is classified as being unmarried (never married, divorced, and widowed) and being married (first married and remarried). Enabling factors included financial status (household income per capita), medical insurance, and social networks (number of local friends). Need factors included self-rated health and number of chronic diseases. The hukou registration type (rural vs. urban) was reported by the participants, which was initially included as a sociodemographic variable but was later used as a grouping variable to investigate whether the determinant factors have different effects on the dependent variables among older adult migrants who have rural or urban hukou registration.

### 2.4. Analysis

First, we described the distribution of all dependent and independent variables, and calculated the rate of receiving free physical examination by different groups. Second, we used the hierarchical random intercept models for binary outcomes [36] to study the impact of contextual and individual variables on the utilization of free physical examinations in the older adult migrants in each city (n = 297). The independence assumption in the single-level multiple regression model is violated when the data are grouped if we didn’t take account of groups effects in our regression model, leading to the underestimation of standard error. Thus, the hierarchical random intercept model for binary outcomes is needed in our analysis to correct the standard error.

**Figure 3:**
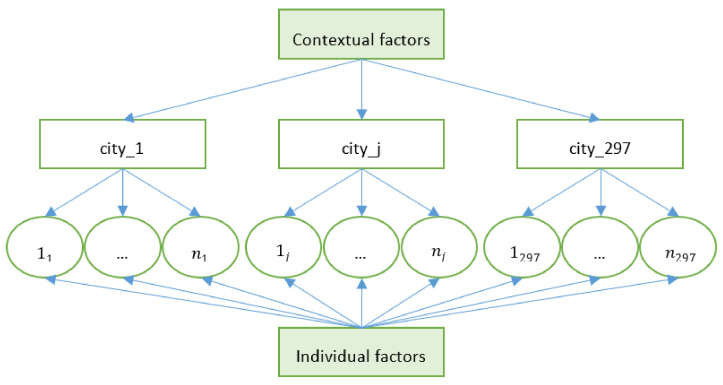
Unit diagram if a two-level nested structure: older migrants in cities.

Assuming a two-level structure, a total of n older adult migrant individuals is nested in group (city) j, of which group (city) j has $n_{j}$ older migrant individuals (Figure 3. Unit diagram if a two-level nested structure: older migrants in cities). We added contextual variables to the city-level equations, which allowed the intercept $\beta_{0j}$ to vary from city to city. By observing the impact of health policy, financing, and organizational factors on the intercept $\beta_{0j}$, we can explore the role of contextual factors in the utilization of BPHS among older adult migrants. When the dependent variable y is 1 (i.e., having received the free medical examination), the logarithmic odds ratio (OR) can be expressed as:

(1) $$log\;\left(\frac{\pi_{ij}}{1-\pi_{ij}}\right)=\gamma_{0}+\beta_{1}predisposing_{ij}+\beta_{2}enabling_{ij}+\beta_{3}need_{ij}+\gamma_{1}policy_{ij}+\;\gamma_{2}financing_{ij}+\gamma_{3}organzation_{ij}+u_{j},$$

where, γ in the equation are coefficients of contextual variables (health policy, financing, and organizational factors), $u_{j}\sim N\left(0,\sigma_{u}^{2}\right)$

The above equation is equivalent to the following two equations:

(2) $$log\;\left(\frac{\pi_{ij}}{1-\pi_{ij}}\right)=\beta_{0j}+\beta_{1}predisposing_{ij}+\beta_{2}enabling_{ij}+\beta_{3}need_{ij},$$

(3) $$\beta_{0j}=\gamma_{0}+\gamma_{1}policy_{ij}+\gamma_{2}financing_{ij}+\gamma_{3}organzation_{ij}+u_{j},$$

## 3. Results

### 3.1. Descriptive Statistics of Individual and Contextual Variables

The descriptive statistics for the individual and contextual variables are presented in Table 2. The mean age was 71.0 (SD = 5.6) and 52.8% were male. Free annual physical examination was received in the past year by 36.2% of total the sample. Those currently married, higher educated, and with urban hukou were more likely to receive free physical examinations. The probability of receiving free physical examinations for older adult migrants living in the host cities for over 3 years and migrating within their hometown province was higher than that of those living in host cities for less than 3 years and migrating across provinces. The proportion of those using BPHS among older adult migrants who had health insurance in their host city was 10% higher than those who did not have health insurance.

### 3.2. Effects of Individual Characteristics

Individual factors had significant impact on the probability of older adult migrants receiving free physical examinations, after controlling for contextual factors.

#### Predisposing Factors

For the total sample, urban hukou was significantly associated with the probability of utilization of BPHS (OR:1.303, CI: 1.123–1.512). Older adult migrants who received middle school or above education were more likely to accept a free physical examination than those who received less education (OR: 1.159, CI: 1.0011–1.342). As for the migrant features, longer living duration in the host cities were positively associated with the utilization of free physical examinations (OR: 1.168, CI: 1.010–1.350), while long relocation distance was negatively associated with the use of BPHS (OR: 0.755, CI: 0.646–0.881).

#### Enabling Factors

Having health insurance in the host cities increased the probability of using the free physical examination (OR: 1.624, CI:1.333–1.979), as did having more friends in the host cities (OR:1.019, CI: 1.009–1.030).

#### Need Factors

Poorer health status had a lower probability of accepting free physical examinations. Compared with the healthy older adult migrants, the probability of accepting the free physical examination for the other two groups was significantly lower. Specifically, the ORs of those with basic health was 0.835 (CI: 0.730–0.954), those who were not healthy was 0.568 (CI: 0.465–0.694). However, having a chronic disease had a higher probability of accepting the free physical examination (OR: 1.292, CI:1.126–1.481).

### 3.3. Effects of Contextual Characteristics

City-level equations in Table 3 reflect the effect of contextual factors (i.e., policy, financing, and organization) on the average level of accepting a free physical examination in each city where the older adult migrants were located. The policy factor was related to the proportion of migrant children enrolled in public schools (OR: 1.024, CI: 1.008–1.041). The organization factor was related to the proportion of urban migrants with established health records, and it positively predicted older migrants receiving free physical examinations (OR: 1.022, CI: 1.016–1.027). GRP, the number of doctors per thousand and the household income of the migrant population per capita in the local area, had no significant effect on free physical examination acceptance.

### 3.4. Difference between the Models for the Rural and Urban Samples

There was no difference in the city-level characteristics between the models for the rural and urban samples, as shown in models 2 and 3 in Table 3. Logistic regression of free medical examination on contextual and individual factors. For the individual-level characteristics, age education and migration distance had a stronger effect for the older adult migrants who had come from rural areas, while duration of living and number of friends in the host city had greater effects for migrants who had come from urban areas.

## 4. Discussion

The rate of using the free annual physical examination among Chinese migrants aged 65 and above was 36.2%, which was lower than that of non-migrant older adults who were living in urban areas (ranged from 54.1% to 84.1%) [27]. The utilization of BHPS among older migrants was so low that the explosion of the determinants of BHPS utilization was worthwhile. Basing on the hierarchical random intercept models, some individual determinants were found as well as contextual characteristics.

### 4.1. Rural-Urban Disparity

Although there were huge differences in various facets between the rural and urban population, and the rate of utilization of free physical examinations was significantly higher among migrant older adults who came from urban compared with rural areas, the contextual and individual characteristics had similar effects on the utilization of BPHS in the two groups. The governments of the host city should pay more attention to older adults arriving from rural areas but should implement similar policies for migrant older adults arriving from either rural or urban areas. Therefore, we will discuss this issue by combining the urban and rural older adult migrants.

### 4.2. Individual Characteristics

The individual factors that affected the use of free physical examinations by older adult migrants included predisposing (socio-demographic factors, migration features), enabling (financial status, health insurance), and need (health status) factors. The major results were consistent with previous studies on the utilization of health services among migrants [15,37].

Among these predisposing factors, the social and migration features showed larger effects on the utilization of BPHS than that of demographic variables. Older adult migrants who had urban hukou, a high level of education, shorter relocation distance, and longer duration of living in their host city were more likely to use the free physical examinations. All of these significant predisposing factors are important variables of social capital, which can help older adult migrants acquire more information about their local BPHS and thus, increase their awareness of their right and pathway to access the BPHS.

Social networks in the destination city as an enabling factor were significantly associated with the likelihood of using the free physical examination, more so than household income or income source. Since the annual physical examination for older adults at a BPHS is free, individual and family economic conditions did not have a significant effect. Instead, it appears that social resources can provide information about public services and promote the utilization of BPHS for migrant populations. This is consistent with our earlier explanation regarding the predisposing characteristics.

Another important individual factor is the need factor, which are measured by the self-report health and chronic conditions. Older adult migrants with chronic diseases were more likely to use the free physical examinations, which is consistent with previous research [27]. However, the relationship between self-rated health status and use of the free physical examination was unexpected albeit was partly consistent with previous research [15]. Older adults with poorer self-rated health status were less likely to use the free physical examination. We once coded the self-rated health as a 4-categroy variable based on the questionnaire, which divided ‘unhealthy’ into ’unhealthy, but do not need help’ and ’need help’. Since the percentage of ’need help’ was small and the coding was confusing, we recoded the self-rated health as a 3-categroy variable. The results didn’t show significant change. A possible explanation is that self-rated health is not the only indicator of health status, but also an indicator of health awareness [38]. Having regular physical examinations is an active and positive health behavior. Kepka and colleagues found that self-rated health was positively associated with health beneficial behaviors in a recently immigrated Latino population [39]. More in-depth analysis (the results can be provided on request) of our data found that the older adult migrants who reported poor health status but did not have a chronic disease had a lower rate of using the free physical examination. Since the condition of chronic diseases were self-reported, the older adults who rated themselves as having poor health but without a chronic illness may not be aware of their possible illness or may have never visited a doctor for their symptoms.

Taken together, the most crucial factors that affect utilization of BPHS at the individual level may be the awareness and information of accepting the free annual examination. Regardless of their financial status, if older adult migrants are aware of their right and the procedures for accessing the BPHS in their destination city as a local resident and understand the health benefits that can be gained by using the BPHS, they are likely to use the BPHS and accept the free physical examinations. Therefore, the key recommendation is to provide more health services information to the older adult migrants, to help them to learn more about the value and accessing approach of BPHS.

### 4.3. Contextual Characteristics

The results for the contextual enabling factors (policy, financing and organization) that influenced BPHS utilization in older adult migrants were not consistent with previous research that focused on other sub-populations. Previous studies have found that, for the general population, the economic development and allocation of health resources in the community was an important prerequisite for individuals to access public health services [29,40]. However, according to the results of two-level hierarchical logistic regression model, the utilization of BPHS by older adult migrants benefitted little from general economic development or increased supply of public services. Only contextual policies and organizational characteristics that relate to migrant population had significant effects on the utilization of BPHS by older adult migrants.

The policies and organizational factors that relate to migrants were positively associated with the use of the free physical examination. In cities where more migrant children attend public schools and there are more migrants with established health records, there was a higher likelihood for the older adult migrants to use the free physical examinations. The proportion of migrant children enrolled in public schools reflects the extent to which the migrant population accesses local public services and thus reflects the city’s policies in support of the migrant population (especially in non-working-age groups). As a result, the proportion of migrant children enrolled in public schools can predict the use of the free physical examinations by older adult migrants. This is evidence for the important role of migrants-supportive policies that allow older adult migrants to access BPHS. The proportion of older adult migrants with established health records had a positive effect on the acceptance of the free physical examinations. Furthermore, the number of doctors for thousand residents in the model was not significant, which indicated that for the older adult migrants, offers of health services that target to the migrant population, instead of the general population, play a positive role in promoting their utilization of BPHS.

The financial factors of the city’s gross domestic product or migrant family household income per capita were not significantly associated with the likelihood for older adult migrants to use the free physical examination. The level of a city’s economic development was not a key factor that restricted access of older adult migrants to BPHS. Although fiscal transfers to local authorities for public service provisions are generally based on the number of hukou residents, the number of older adults participating in free physical examinations is relatively small, so most of the communities and cities can currently afford the cost of the BPHS [41].

### 4.4. Limitations

There are several limitations of this study that must be noted. First, the data came from a cross-sectional survey, so trends or long-term effects on older migrants’ BPHS utilization could not be explored. Secondly, when discussing the difference between urban and rural hukou, the sample size in some cities was small (e.g., only one or two cases), which might have affected the stability of the models. Thirdly, due to the limited data, we did not include all the contextual influencing factors in the study, which warrant further exploration.

## 5. Conclusions

The bottleneck in accessing the BPHS by older adult migrants at the contextual level is not the destination cities’ lack of financial input or health resources, but the acceptance and inclusion of the migrants in society. Only policies that benefit the migrant population have real impetus. At the individual level, the awareness and information regarding the value and access of BPHS are the major factors that influence the utilization of BPHS in older adult migrants.

This study offers policy implications for China’s ongoing equalization of the BPHS. To increase the proportion of older migrants to use the free physical examination, promote the equalization of basic public health services, and achieve overall healthy aging, the local government should eliminate policy barriers, and target the migrant population in promoting urban services. Although older adult migrants are included in the target population of local public health services, the publicity and education of health service-related knowledge in this group need to be strengthened. This would allow all older adults, including migrants, to fully appreciate the benefits that can be gained from the BPHS.

## Figures and Tables

**Table 1:** Contextual (city-level) variables: Health policy, financing and organization.

Elements	Dimensions	Indicators	Specific Variables
Policy	General	--	--
	Migrant related	Accessibility of public services for the migrant population	Proportion of migrant children enrolled in public schools (%)
Financing	General	general economic situation of the city	Gross regional product (GRP) per capita (thousand Yuan)
	Migrant related	Economic situation of the migrant population	Household income of the migrant population per capita (thousand Yuan)
Organization	General	Health resource allocation	Number of practicing physicians per 1000 people (person)
	Migrant related	Health resources of the migrant population	Proportion of migrant population with health record (%)

**Table 2:** Individual and contextual variables, and the associations with free examinations.

Variables	Values	n	Mean (SD)/%	Free Examination (%)
Predisposing				
Age	65–69	2933	49.9	51.6
	70–79	2391	40.7	39.7
	80+	552	9.4	8.7
Sex	Male = 0	3101	52.8	36.3
	Female = 1	2775	47.2	36.0
Education	Primary school or lower = 0	3737	63.6	34.8
	Middle school and above = 1	2139	36.4	38.6
Marital status	Unmarried = 0	1498	25.5	32.1
	Married = 1	4378	74.5	37.5
Hukou	Rural = 0	3680	62.6	33.8
	Urban = 1	2196	37.4	40.0
Living duration	<3 years = 0	1408	24.0	33.2
	≥3 years	4468	76.0	37.1
Migration scope	Within province = 0	3500	59.6	40.4
	Across province = 1	2376	40.4	29.8
Enabling				
Family income	(0–28,000)	5876	1813.67 (1439.45)	
Medical insurance	No = 0	5258	89.5	34.7
	Yes = 1	618	10.5	48.2
Friends number	(0–21)	5876	6.8 (6.1)	
Need				
Self-rated health	Healthy = 1	2065	35.1	37.8
	Basically healthy = 2	2872	48.9	36.8
	Unhealthy = 3	939	16.0	30.5
Chronic conditions	No = 0	4238	72.1	35.4
	Yes = 1	1638	27.9	38.0
Children enrolled	(49.8, 99.7)	5876	90.2 (9.3)	
Gross regional product	(8.4, 196.73)	5876	61.6 (31.3)	
Household income	(0, 50)	5876	6.2 (3.2)	
Practicing physicians	(0.87, 4.42)	5876	2.5 (0.6)	
Health record	(0, 97.5)	5876	30.9 (22.1)	

Notes: Children enrolled indicates Proportion of migrant children enrolled in public schools (%): Gross regional product indicates Gross regional product (GRP) per capita (thousand Yuan); Household income indicates Household income of the migrant population per capita (thousand Yuan); Practicing physicians indicates Number of practicing physicians per 1000 people (person); Health record indicates Proportion of migrant population with health record (%).

**Table 3:** Logistic regression of free medical examination on contextual and individual factors.

	(1) Overall	(2) Rural Origin	(3) Urban Origin
VARIABLES	OR (*95% CI*)	OR (*95% CI*)	OR (*95% CI*)
Individual-level characteristics			
Predisposing			
Age centered (at age 70)	0.991	0.980 ***	1.002
	(0.979, 1.002)	(0.965, 0.995)	(0.984, 1.021)
Female	1.070	1.051	1.068
	(0.947, 1.209)	(0.896, 1.233)	(0.880, 1.297)
Married	1.132	1.134	1.066
	(0.971, 1.320)	(0.940, 1.369)	(0.805, 1.413)
Middle school and above	1.159 **	1.263 **	1.010
	(1.001, 1.342)	(1.029, 1.551)	(0.812, 1.256)
Urban hukou	1.303 ***		
	(1.123, 1.512)		
Living duration >3 years	1.168 **	1.070	1.280 **
	(1.010, 1.350)	(0.890, 1.288)	(1.005, 1.630)
Migrating across province	0.755 ***	0.700 ***	0.866
	(0.646, 0.881)	(0.576, 0.852)	(0.664, 1.129)
Enabling			
Avg household income (log)	1.038	1.026	1.062
	(0.970, 1.111)	(0.947, 1.110)	(0.917, 1.230)
Insured at destination	1.624 ***	1.573 ***	1.637 ***
	(1.333, 1.979)	(1.221, 2.026)	(1.170, 2.290)
Friends number at destination	1.019 ***	1.006	1.032 ***
	(1.009, 1.030)	(0.992, 1.021)	(1.016, 1.047)
Need			
Self-rated health (healthy = ref.)			
Basically healthy	0.835 ***	0.893	0.780 **
	(0.730, 0.954)	(0.747, 1.068)	(0.632, 0.963)
Unhealthy	0.568 ***	0.554 ***	0.611 ***
	(0.465, 0.694)	(0.431, 0.712)	(0.431, 0.865)
Having chronic disease	1.292 ***	1.289 ***	1.261 **
	(1.126, 1.481)	(1.072, 1.550)	(1.021, 1.558)
Constant	0.0108 ***	0.0181 ***	0.0256 ***
	(0.00187, 0.0626)	(0.00238, 0.138)	(0.00178, 0.367)
City-level characteristics			
Policy, financing, and organization			
Migrant children enrolled in public school	1.024 ***	1.023 **	1.021 *
	(1.008, 1.041)	(1.004, 1.041)	(0.997, 1.044)
GRP per capita	0.997	0.997	0.998
	(0.993, 1.002)	(0.991, 1.002)	(0.992, 1.004)
Migrant household income per capita	0.963	0.956	0.969
	(0.919, 1.010)	(0.902, 1.013)	(0.911, 1.031)
Practicing physicians per 1000	1.176	1.219	1.106
	(0.934, 1.482)	(0.936, 1.589)	(0.819, 1.492)
Migrant with health record	1.022 ***	1.023 ***	1.017 ***
	(1.016, 1.027)	(1.017, 1.030)	(1.009, 1.026)
Variance (_cons[city])	1.765 ***	1.962 ***	1.642 ***
	(1.431, 2.177)	(1.487, 2.588)	(1.205, 2.239)
Observations	5876	3680	2196
Number of groups	297	288	191

Notes: OR = Odds ratio; CI = Confidence interval; *** *p* < 0.01, ** *p* < 0.05, * *p* < 0.1.

## Data Availability

Restrictions apply to the availability of these data. Data was obtained from Migrant Population Service Center, National Health Commission P. R. China and are available from the authors with the permission of Migrant Population Service Center, National Health Commission P. R. China
